# High temperature deformability of ductile flash-sintered ceramics via in-situ compression

**DOI:** 10.1038/s41467-018-04333-2

**Published:** 2018-05-25

**Authors:** Jaehun Cho, Qiang Li, Han Wang, Zhe Fan, Jin Li, Sichuang Xue, K. S. N. Vikrant, Haiyan Wang, Troy B. Holland, Amiya K. Mukherjee, R. Edwin García, Xinghang Zhang

**Affiliations:** 1 School of Materials Engineering, Purdue University, West Lafayette, IN 47907 USA; 2 Oak Ridge National Laboratory, Oak Ridge, TN 37830 USA; 3 School of Electrical and Computer Engineering, Purdue University, West Lafayette, IN 47907 USA; 4 Department of Mechanical Engineering, Colorado State University, Fort Collins, CO 80523 USA; 5 Department of Chemical Engineering & Materials Science, University of California, Davis, CA 95616 USA

## Abstract

Flash sintering has attracted significant attention as its remarkably rapid densification process at low sintering furnace temperature leads to the retention of fine grains and enhanced dielectric properties. However, high-temperature mechanical behaviors of flash-sintered ceramics remain poorly understood. Here, we present high-temperature (up to 600 °C) in situ compression studies on flash-sintered yttria-stabilized zirconia (YSZ). Below 400 °C, the YSZ exhibits high ultimate compressive strength exceeding 3.5 GPa and high inelastic strain (~8%) due primarily to phase transformation toughening. At higher temperatures, crack nucleation and propagation are significantly retarded, and prominent plasticity arises mainly from dislocation activity. The high dislocation density induced in flash-sintered ceramics may have general implications for improving the plasticity of sintered ceramic materials.

## Abstract (web summary)

Flash sintering allows for rapid ceramic processing, but the mechanical behavior of such ceramics remains poorly understood. Here, the authors compress micropillars of yttria stabilized zirconia to show flash sintering promotes outstanding plasticity.

## Introduction

Ceramic materials have a variety of high-temperature applications, such as thermal barrier coatings for high-thrust engines and turbines^1^. One promising candidate for thermal barrier coatings is yttria-stabilized zirconia (YSZ). YSZ offers one of the lowest thermal conductivities, ~2.3 W/mK at ~1000 °C^2^. Monolithic zirconia has monoclinic phase (space group *P*2_1_/*c*) at room temperature. However, the tetragonal phase (space group *P*4_2_/*nmc*) of zirconia can be stabilized by doping zirconia with Y_2_O_3_, CeO_2_, and MgO^3,4^. The discovery of martensitic phase transformation (from tetragonal to monoclinic phase) in ZrO_2_ has led to significant investigations on its deformability. The volume expansion (~4%) during martensitic phase transformations near the crack tips induced by an external stress can introduce compressive stress that can in turn retard crack propagation^5,6^. Therefore, YSZ provides new opportunities for various applications, including reliable thermal and environmental barrier coatings, solid oxide fuel cells, and shape memory devices, just to name a few^1,7,8^.

A majority of ceramic materials possess high strength but low toughness at low temperatures due to the lack of dislocation-enabled deformability^9^. Certain nanostructured ceramics have shown high strength, wear resistance, and/or fracture toughness at elevated temperatures^10–12^. However, conventional sintering typically requires very high temperature and long sintering time and thus leads to significant grain coarsening^13^. Recently, it has been discovered that YSZ can be fully densified within a few seconds at a temperature much lower than conventional sintering temperature by a novel sintering technique named flash sintering^14^. The ultrafast sintering technique enables the retention of nanograins and enhanced dielectric properties^15^. Flash sintering occurs by applying a ramp heating process at a constant heating rate under moderate electrical fields^16–24^. Once the temperature is above the onset of the flash temperature, under applied electrical field, a densification process takes place within a few seconds as evidenced by a sudden increase of electrical conductivity, accompanied with drastic increase in mass density^25^.

Prior studies on the mechanical behavior of YSZ showed superelasticity and shape memory effect at room temperature^26–30^. However, our understanding of the mechanical behaviors of small-scale YSZ specimens at elevated temperatures remains limited. Recently, Korte and Clegg showed that microcompression tests on small specimens could be carried out at an elevated temperature (~500 °C) by heating the sample stage and indenter tip without a significant mechanical and thermal drift (~1 nm/s)^31,32^. High-temperature micropillar compression technique enables the study on the temperature-dependent deformation mechanisms for brittle materials at elevated temperatures. Furthermore, the deformability of flash-sintered ceramics is largely unknown despite their intriguing microstructures, including the generation of a large number of charged defects during the flash-sintering process^25^.

Here, we report an in situ micropillar compression study on the deformability of a flash-sintered 3 mol% yttria stabilized zirconia (3YSZ) at elevated temperatures (up to 600 °C). The flash-sintered 3YSZ contains abundant dislocations. The mechanical behaviors of the flash-sintered 3YSZ are highlighted by increased plasticity, and temperature-dependent transition of deformation mechanisms.

## Results

### Microstructural characterization

**Fig. 1:**
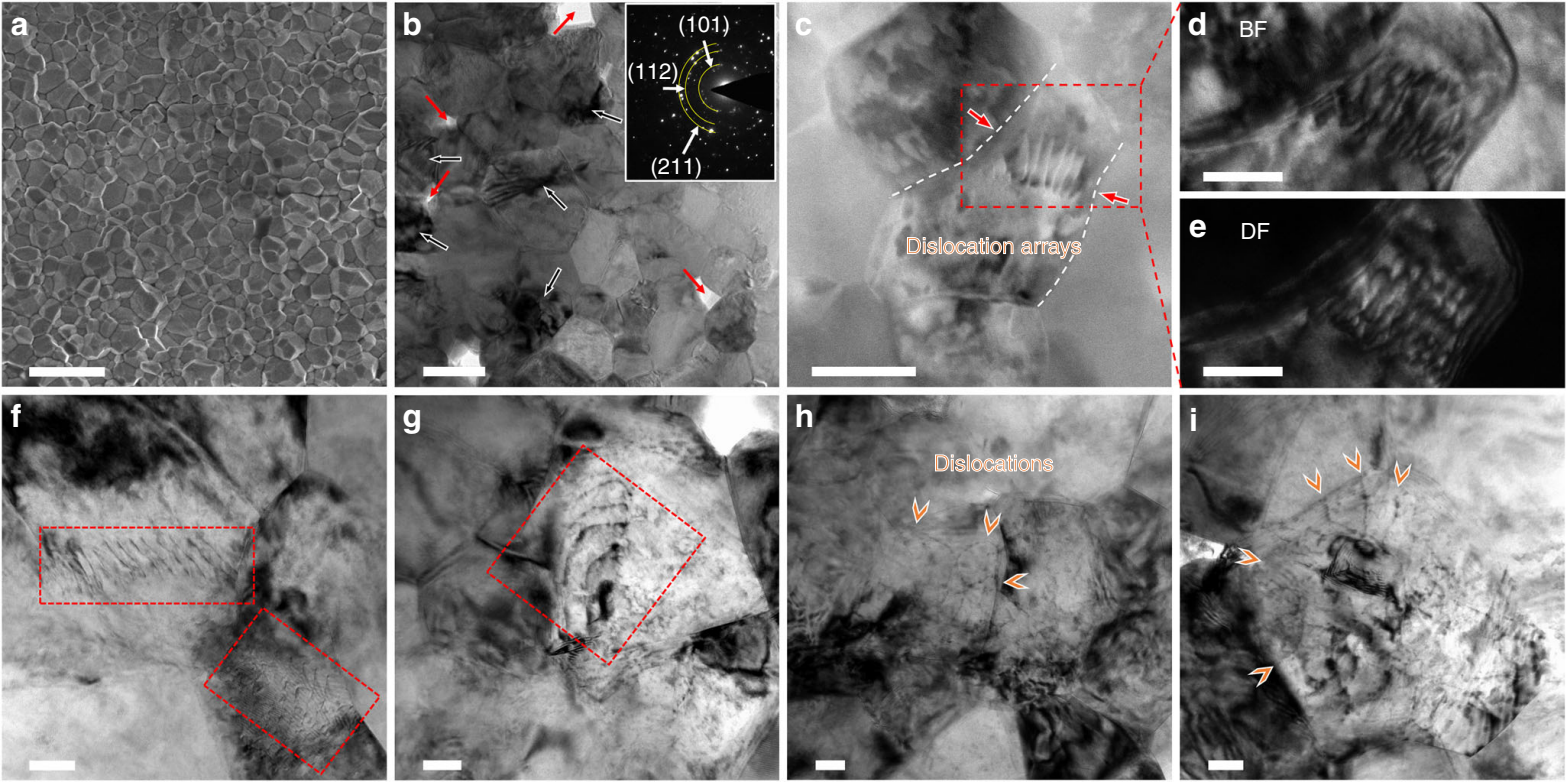
Microstructure of flash-sintered 3YSZ. **a** SEM images of the unpolished flash-sintered 3YSZ. The average particle size is ~1 μm. Scale bar, 3 μm. **b** Bright-field TEM micrograph showing subgrains with grain boundaries and defects generated during flash sintering (labeled by black arrows), and nanopores (~1.4%) (indicated by red arrows). The inserted SAD pattern shows diffraction rings. Scale bar, 200 nm. **c** A STEM micrograph showing a dislocation array inside a grain in the flash-sintered 3YSZ. The dislocation array was generated in a bottleneck region of the grain as indicated by red arrows. Scale bar, 100 nm. **d**, **e** Bright-field (BF) and dark-field (DF) TEM micrographs showing the dislocation array in the boxed region in **c**. Scale bar, 50 nm. **f**–**i** Numerous BF TEM images of the flash-sintered 3YSZ showing the existence of dislocations and dislocation arrays in grains. Scale bar, 50 nm

3YSZ (TZ-3Y-E, Tosh corp., 40 nm) was heated in a thermomechanical analyzer with platinum electrodes at a constant heating rate of 25 °C/min under an electrical field of 150 V/cm. The ultrafast densification process occurred at a furnace temperature of 1150–1200 °C in a few seconds (Supplementary Fig. 1), which is significantly lower compared to conventional sintering temperature of ~1900 °C to sinter 3YSZ in just a few seconds^33^. The applied heat and electrical field were removed right after the onset of flash sintering to prevent grain growth. Even though the densification process of 3YSZ occurred at relatively low temperature, X-ray diffraction pattern shows a dominant tetragonal phase without evident monoclinic and cubic phases (Supplementary Fig. 2). Energy dispersive spectroscopy reveals that zirconium and yttrium are uniformly distributed throughout the grains, and zirconium is slightly deficient along grain boundaries (Supplementary Fig. 3). Figure 1a shows a scanning electron microscopy (SEM) image of an unpolished flash-sintered 3YSZ. An average grain size of 870 nm was determined by a systematic grain intercept method^34^ (Supplementary Fig. 4). However, bright-field transmission electron microscopy (TEM) micrograph reveals the existence of subgrains as shown in Fig. 1b. The average subgrain size is 159 nm. The flash-sintered 3YSZ was densified to 98% of theoretical density with nanopores indicated by red arrows in Fig. 1b. Black arrows in Fig. 1b indicate internal defects in grains generated during the flash sintering process. Deformation twinning which occurred frequently in bulk tetragonal zirconia^35^ was rarely observed in this study, presumably due to the ultrafine grain sizes.

### Preexisting dislocations

Many of the defects in the grains are identified to be high-density dislocation networks (as shown in Supplementary Fig. 5). Furthermore, scanning TEM (STEM), bright-field and dark-field TEM micrographs in Fig. 1c–e show an array of dislocations inside a grain. Also, the dislocation arrays were observed frequently in the flash-sintered 3YSZ as shown in Fig. 1f–i and Supplementary Fig. 6. Meanwhile, 3YSZ synthesized at lower electric fields (1.5 and 15 V/cm) and at the same heating rate (25 °C/min) was also prepared to study the effect of electric field on the dislocation generation. The relative density of 78% and 85% was achieved for the 1.5 V/cm and 15 V/cm specimens, respectively (Supplementary Fig. 1). The grain intercept method^34^ reveals average grain sizes of 59±7 nm and 57±9 nm for 3YSZ sintered under 1.5 V/cm and 15 V/cm, respectively. The TEM studies reveal little dislocations or dislocation arrays in these specimens (Supplementary Fig. 7).

### In situ microcompression tests

**Fig. 2:**
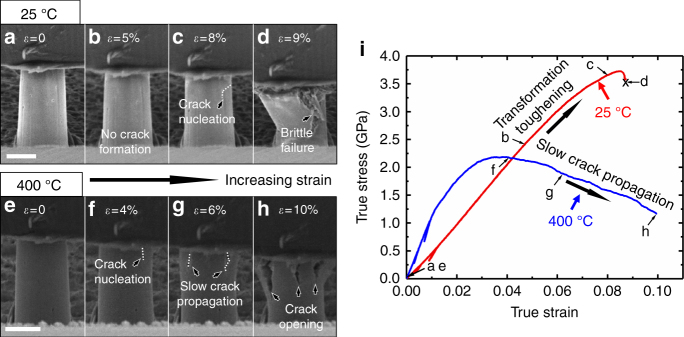
Uniaxial in situ microcompression tests on the flash-sintered 3YSZ at room temperature and 400 °C at a constant strain rate of 5 × 10^−3^ s^−1^. **a**–**d** SEM images during in situ compression test of a micropillar at different strain levels at 25 °C. No crack can be detected until a true strain of 8%. At the strain of ~9%, the pillar experienced brittle catastrophic fracture. Scale bar, 2 μm. **e**–**h** For micropillars tested at 400 °C, cracks nucleated at smaller strain, ~4%. Crack density increased with compressive strain. However, cracks propagated downward gradually and slowly without catastrophic failure. Scale bar, 2 μm. **i** The corresponding true stress–strain curve shows that the flow stress exceeds 3.5 GPa for pillars tested at 25 °C. In comparison, the pillar tested at 400 °C has a flow stress of 2 GPa and higher elastic modulus determined from partial loading and unloading experiments

Micropillars with ~3 μm in diameter and ~6 μm in height were fabricated using the focused ion beam (FIB) technique on the flash-sintered 3YSZ. Given that the average subgrain size of the specimen is ~160 nm, each pillar contains more than 5000 subgrains. Thus, the mechanical behaviors of the micropillars are a good representation of a large number of grains. Uniaxial in situ microcompression tests on the micropillars were carried out from room temperature to 600 °C at a constant strain rate of 5 × 10^−3^ s^−1^ inside an SEM microscope with partial unloadings at strains of 0.5 and 1%, to evaluate the apparent elastic modulus at each test temperature (Supplementary Movies 1–3). Figure 2 shows snapshots of SEM images taken during the in situ compression tests of micropillars at different strain levels at room temperature and 400 °C. Micropillars compressed at room temperature sustained a strain up to ~8% without crack formation, and then experienced brittle catastrophic fracture at a strain of ~9%. For micropillars tested at 400 °C, cracks nucleated at smaller strain, ~4%, and a greater crack density was observed when compared to the specimens tested at room temperature. Meanwhile, cracks propagated downward gradually along the axial (loading) direction, but no catastrophic failure was observed. Figure 2i shows that the maximum flow stress exceeds 3.5 GPa in specimens tested at room temperature; whereas the peak stress reaches 2 GPa for specimens tested at 400 °C at a true strain of 4%, and the stress decreases thereafter.

### Temperature dependence of deformation mechanisms

**Fig. 3:**
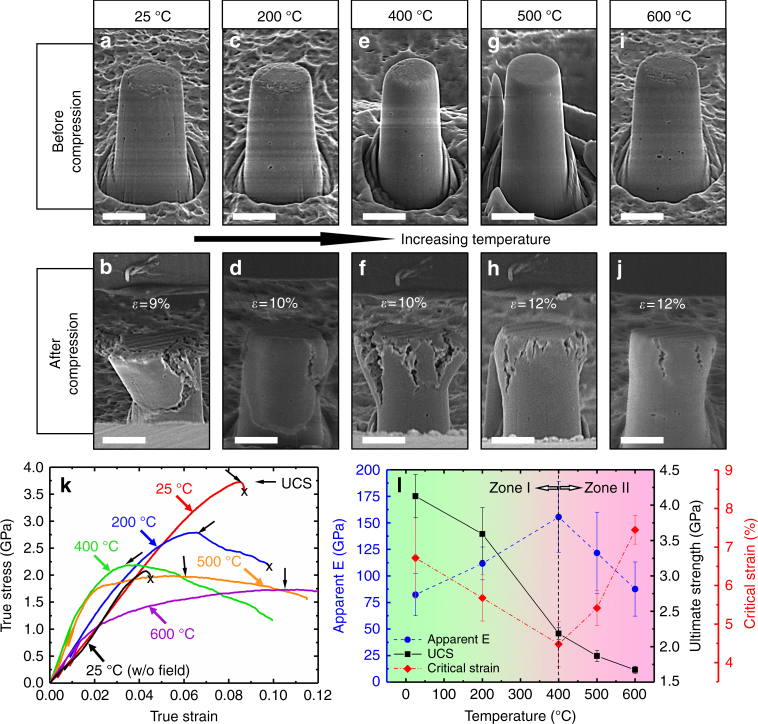
SEM images of the flash-sintered 3YSZ micropillars before and after compression tests from 25 to 600 °C and corresponding mechanical properties. **a**–**d** When tested at 200 °C and below, the pillar fractured in a brittle manner (into two major sections) at very large true strain. Scale bar, 2 μm. **e**–**j** When tested at 400–600 °C, multiple cracks formed and propagated slowly into the pillars, leading to formation of the cauliflower type of pillar tops. At 600 °C, crack density and propagation distance were substantially reduced. Scale bar, 2 μm. **k** Corresponding true stress–strain curves of pillars tested at different temperatures. Black arrows indicate the ultimate compressive strength (UCS) of the pillars. Partial unloadings at 0.5 and 1% strains were performed to investigate the apparent elastic moduli of pillars tested at different temperatures. A stress–strain curve for 3YSZ sintered without the electrical field is shown as reference. **l** Ultimate compressive strength (UCS) decreases monotonically with increasing test temperature. The elastic modulus increases with test temperature up to 400 °C and decreases thereafter. Meanwhile, the critical strain at which the first crack nucleates decreases with temperature to a minimum of 4.5% at 400 °C and increases thereafter to 7.5% when tested at 600 °C. 400 °C is the onset temperature where a different inelastic deformation mechanism begins to operate. Zone 1 represents phase transformation toughening from room temperature to 400 °C. Zone 2 corresponds to dislocation creep dominant plasticity above 400 °C

Figure 3a–j compares SEM images of flash-sintered 3YSZ micropillars before and after microcompression tests at various temperatures. It shows that 400 °C is a fiducial temperature at which fracture mechanisms change drastically. At 25 and 200 °C, the micropillars, though they sustained a large strain, fractured in a brittle (catastrophic) manner. On the other hand, when tested between 400 to 600 °C, multiple cracks initiated and propagated from the top surface down into the pillars, leading to the formation of cauliflower morphologies at the top of the pillars (Fig. 3f, h, j). As the test temperature rose, a prominent decrease of crack density and propagation was observed, implying that a new deformation mechanism began to govern the inelastic behavior of the pillars at 400 °C and beyond. Figure 3k compares corresponding stress–strain curves of pillars tested at different temperatures with black arrows, indicating the ultimate compressive strength (UCS) of the pillars. Five true stress-true strain curves were obtained for reproducibility tests at each temperature (shown in Supplementary Fig. 8). The UCS of the flash-sintered 3YSZ decreases monotonically with increasing test temperature as shown in Fig. 3l. However, elastic modulus (measured from a series of partial unloading experiments) first increases with test temperature up to 400 °C and decreases thereafter. The critical strain for the nucleation of cracks decreases with increasing test temperature, reaches a minimum at 400 °C, and then increases to ~7.5% at 600 °C. Meanwhile, 3YSZ was also sintered without electric field at a constant heating rate of 25 °C/min to 1300 °C and exhibited a failure stress of 2 GPa and a failure strain of 4%.

### Martensitic transformations

Meanwhile, we carried out TEM experiments on the YSZ pillars after microcompression tests tested at room temperature. Supplementary Fig. 9a–b shows the SEM micrographs of the pillar before and after compression test at room temperature. The pillar fractured at a flow stress of ~4 GPa and a strain of ~6% (Supplementary Fig. 9c). Bright-field XTEM image of the fractured pillar clearly shows the fracture surface and embedded ultrafine grains (Supplementary Fig. 9d–f). Numerous grains in vicinity of the fracture surface were examined carefully. The diffraction pattern of grain 1 (Supplementary Fig. 9g) obtained along the [$\bar 101$] zone axis was indexed to be a monoclinic phase (JCPDS#37-1484). The interplanar spacing of the (111) planes is measured to be 0.279 nm, consistent with the theoretical value of 0.284 nm. Grains 2 and 3 were examined along respective zone axis of [$1\bar 32$] and [$\bar 112$] and indexed to be monoclinic zirconia phase. These studies show that the tetragonal-to-monoclinic phase transition indeed took place during the compression tests.

### Cyclic loading tests

**Fig. 4:**
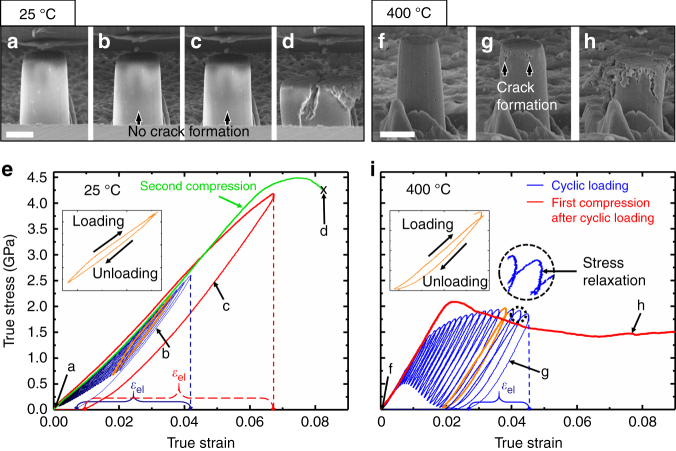
Cyclic loading and subsequent monotonic compression tests at a strain rate of 5 × 10^−3^ s^−1^ at 25 and 400 °C and the corresponding stress–strain curves. **a**, **f** SEM images of micropillars before cyclic loading tests. Scale bar, 2 μm. **b**, **g** SEM micrographs of micropillars after 30 cyclic loading tests. The cyclic stress–strain curves are shown as blue curves in **e** and **i**. **c**, **h** After the first monotonic compression tests highlighted in red curves. **d** After the second monotonic compression test highlighted in green. The 27th loading–unloading curves at each temperature shown in orange color and enlarged in the inserted stress–strain curves clearly show the hysteresis loops. The upper portion of a loading–unloading curve is enlarged in a circle to illustrate the stress relaxation for 1 s of holding at 400 °C

Thirty cyclic loading and partial unloading tests were carried out at a strain rate of 5 × 10^−3^ s^−1^ at room temperature and 400 °C as shown in Fig. 4. Holding segments prior to unloading for 1 s (during which displacement remains constant) were added. First, 30 cyclic loadings (with increasing strain in each cycle) up to a strain of 4% were conducted with partial unloading to half of the maximum applied strain in each cycle in order to maintain a solid contact between the diamond tip and pillar. No crack was observed for the specimens tested at room temperature during the cyclic tests, and the pillar exhibited a significant amount of recoverable strain during unloading (Fig. 4b). After cyclic tests, a monotonic compression test (highlighted by a red curve in Fig. 4e) was conducted on the same micropillar up to a strain of 7%. The pillar exhibited ~6% recoverable and ~1% irrecoverable strain without crack (Fig. 4c). The same pillar then experienced a catastrophic failure in a succeeding monotonic compression test (Fig. 4d) at a strain of 8%, shown by a green curve in Fig. 4e. In comparison, YSZ subjected to cyclic loading tests at 400 °C (Fig. 4i) showed much less recoverable strain than that tested at room temperature. Also, small cracks formed (as shown in Fig. 4g) at a strain of 4% during the cyclic loading test. The subsequent monotonic microcompression test on the same pillar highlighted as a red curve in Fig. 4i shows that the pillar exhibited a UCS of 2 GPa, and a residual strength of 1.5 GPa at a strain of 9%, and multiple cracks formed after compression tests (Fig. 4h).

## Discussion

In general, dislocations are rare in ceramics as the strong covalent and ionic bond greatly discourages the formation of dislocations. However, TEM studies herein show ample evidence for the formation of high-density dislocations in flash-sintered 3YSZ. Also, dislocation arrays are frequently observed near triple junctions. It is likely that a large stress concentration (gradient) develops near triple junctions during the rapid grain growth process. Significant mass (atomic) transport occurs during the flash-sintering process to fill in the voids among grains/particles during a very short sintering time (several seconds). The high rate (4–5 orders of magnitude faster than conventional sintering) of mass transport and flow near triple junctions may lead to substantial plastic deformation (enabled by high-density dislocation arrays as shown in Fig. 1c) during flash sintering^25^. This assertion coincides with TEM studies of 3YSZ sintered under 1.5 and 15 V/cm, which display insignificant grain growth and little dislocations. Rapid grain growth and large electric field may play a significant role in producing dislocations and their arrays by generating the internal stress during flash sintering. The electric field of 1.5 and 15 V/cm is insufficient to induce dislocations in 3YSZ. Interestingly, intragranular dislocations and dislocation entanglement in 3YSZ have been observed at an ultrahigh temperature, 1400 °C, during tensile creep tests at 50 MPa^36^. In the current study, the generation of intragranular dislocations, their arrays and entanglement near triple junctions may strongly depend on stress and high strain rate plastic flow during flash sintering. The shear stresses allowing dislocation pile-up in 3YSZ range from 350 to 1260 MPa based on the lattice dislocation pile-up model^37^:

1 $${\mathrm{\tau }} = \frac{{Gb}}{{2L}}N$$

where *G* is the shear modulus, *b* is the Burgers vector, *N* is the number of intragranular dislocations in the pile-up within a grain, and *L* is the length of dislocation pile-ups. From TEM measurement of dislocation separation distances in the pile-ups, ~20 nm, in flash-sintered 3YSZ, the shear stress during flash sintering is estimated to be ~1230 MPa (by taking *G* = 65 GPa and *b* for <110> type lattice dislocation = 3.6 × 10^−10^ m), which is within the range that can form dislocation arrays in 3YSZ at elevated temperatures during flash sintering. The dislocation density in many grains reaches as high as ~2–3 × 10^12^ m^−2^, comparing to the typical density of ~10^8^–10^10^ m^−2^ in a majority of ceramics^38^. These high-density dislocations play a vital role on the deformability of the flash-sintered 3YSZ tested at elevated temperatures. It is well known that dislocation glide is unlikely to take place in bulk ceramic materials at room temperature unless applying a confinement pressure via hydrostatic or gaseous medium^31^. However, at microscales, plastic deformation of certain ceramics by glide of dislocations has been observed^39,40^. Camposilvan et al. investigated the plasticity of conventionally sintered YSZ at small scales at room temperature and speculated that dislocation activity along with transformation-induced plasticity at a higher stress level (without showing direct evidence of dislocations) may be a possible inelastic deformation mechanism for YSZ^28^. Our finite-element method analysis on stress distribution for dislocations in polycrystalline YSZ further supports this assertion and shows that shear stress concentration near-grain boundaries and triple junctions induces the nucleation and migration of dislocations, thereby facilitating the plastic deformation of YSZ (Supplementary Figs. 10 and 11 and Supplementary Methods).

A strain of ~8% for stabilized zirconia owing to martensitic transformation-induced plasticity has been previously reported^8,27–30^. However, micropillars exhibiting such a large strain were often limited to single and oligocrystalline structures to minimize internal mismatch stresses during martensitic transformation. The micropillars of the flash-sintered 3YSZ consist of subgrains, ~160 nm in diameter, much smaller than the diameter of the micropillars (3 μm). Thus, the large ~8% inelastic flow may arise from not only transformation-induced plasticity, but also dislocation activity especially at higher stress level.

For micropillars tested at 400 °C, cracks nucleated at smaller strain, ~4%, due to the lack of martensitic transformation toughening. At elevated temperatures, the metastable tetragonal phase begins to thermally transform into a mixture of tetragonal and cubic phase, degrading the deformability of the flash-sintered 3YSZ by transformation toughening^37^. However, at 400 °C, cracks initiated from the top surface of the micropillar propagate downward gradually and slowly without catastrophic failure unlike the brittle catastrophic fracture of micropillars tested at room temperature. The prominent variation of fracture morphology of the deformed pillars implies that a new inelastic deformation mechanism supersedes martensitic transformation toughening as temperature rises. In the conventionally sintered bulk YSZ system, it is known that the 700–800 °C temperature range favors other mechanisms (grain boundary sliding, ferroelastic domain switching, and/or dislocation activity) as a substitute to martensitic transformation^41^. However, the flash-sintered YSZ contains nanograins, oxygen vacancies^15^, and abundant preexisting dislocations, which may result in the early activation of other inelastic deformation mechanisms at 400 °C^42^. It is worth mentioning that the critical strain for the pillars compressed at 200 °C is still high (~6%). Catastrophic failure of pillars was also observed for specimens tested at 200 °C, and transformation toughening-induced plasticity remains the dominant inelastic deformation mechanism of the flash-sintered 3YSZ.

At an even greater test temperature, the martensitic phase transformation toughening is gradually replaced by the activation of a new inelastic deformation mechanism. Basically, the critical strain at which the first crack occurs decreases with increasing test temperature and reaches a minimum at ~400 °C as can be seen in Fig. 3i. The earlier occurrence of cracks at an elevated temperature (at lower critical strain than that at room temperature) implies a lack of transformation toughening. The critical strain for the onset of crack propagation increases when the test temperature is >400 °C. Furthermore, in contrast to the crack-triggered catastrophic failure in low-temperature specimens (25 and 200 °C), cracks in high-temperature specimens (≥400 °C) propagate slowly and are more uniformly distributed in the top portion of the deformed pillar, leading to the dilated cauliflower morphology. The slow crack propagation speed indicates enhanced compressive ductility at elevated temperature. As phase transformation toughening is less likely to operate at high temperatures, the enhanced plasticity of YSZ may arise from deformation mechanisms, such as dislocation creep and/or grain boundary sliding. The high-density dislocations in flash-sintered specimens suggest that dislocation power creep type of mechanism is highly likely. Meanwhile, the small grains retained in the flash-sintered specimens may promote grain boundary sliding as a favorable deformation mechanism. Therefore, 400 °C is the brittle-to-ductile transition temperature for flash-sintered YSZ, a much lower value than the one reported for conventionally sintered or single-crystal YSZ systems (~800 °C)^43^.

The apparent elastic moduli of tested YSZ are lower than the theoretical values (210 GPa) at all test temperatures. It is well known that the underestimation of elastic modulus in the microcompression tests can be attributed to taper angle of the pillars, misalignment between the tips and pillars, and stress concentration on the top surface of the pillars^28^. Taking the underestimation into consideration, it is still surprising that the apparent elastic modulus of the flash-sintered 3YSZ increases with temperature and reaches a maximum at 400 °C (Fig. 3i). This is because: (a) the dominant phase of 3YSZ at room temperature is metastable tetragonal phase of zirconia (Supplementary Fig. 2) and the elastic modulus of tetragonal phase is known to be lower than that of monoclinic and cubic phase^44,45^. When temperature increases, an increasing portion of tetragonal zirconia undergoes transformation to stable tetragonal and cubic phase, which may lead to the slight increase of the apparent elastic modulus^46^. (b) Ceramic materials retaining superelasticity usually exhibit lower elastic modulus due to the strain burst. It follows that the larger elastic modulus observed at elevated temperatures is an indication of suppression of the martensitic phase transformation due to thermodynamically reinforced stability of tetragonal phase^47,48^. The total free energy change for martensitic transformation per unit volume (Δ*U*_0_) can be expressed as^47^,

2 $$\Delta U_0 = \Delta U_{\mathrm{c}} + \Delta U_{\mathrm{e}} + \Delta U_{\mathrm{s}} - \Delta U_{\mathrm{I}}$$

where Δ*U*_c_ is the chemical free energy change, Δ*U*_e_ the elastic energy change associated with volume expansion of tetragonal zirconia constraint by matrix, Δ*U*_s_ the interface energy change (negligible), and Δ*U*_*I*_ the change in free energy associated with an external applied stress. This mechanical term should be larger than the first three terms on the right-hand side for martensitic transformation to occur. When the chemical free energy term ($\Delta S_{\mathrm{m}}(M_{\mathrm{s}} - T)$) and additional free energy change ($\Delta S_{\mathrm{m}}(M_{\mathrm{s}} - T_{\mathrm{0}})$) due to the presence of dopant are taken into account^48^, the total free energy change as a function temperature is given by,

3 $$\Delta U_{\mathrm{0}} = \Delta S_{\mathrm{m}}\left( {M_{\mathrm{s}} - {T}} \right) - \Delta U_{\mathrm{I}}$$

where $\Delta S_{\mathrm{m}}$ is entropy change associated with martensitic transformation and *M*_s_ is the martensitic start temperature. When *M*_s_ = *T*, spontaneous martensitic transformation takes place without the external stress. As test temperature increases, Eq. (3) shows that the chemical energy change also increases (note that entropy term is negative value), thereby requiring a larger mechanical term to overcome free energy barrier for martensitic transformation to occur.

The higher temperature (*T* > 400 °C) weakens the interatomic bonds and thus reduces the elastic modulus, and high-density flash-sintered dislocations significantly contribute to the plasticity of 3YSZ as manifested by the increasing critical strain to nearly 7.5% before the observation of cracks at 600 °C. In summary, elastic modulus, critical strain, and fracture behavior of flash-sintered 3YSZ at each test temperature indicate that 400 °C is the temperature beyond which inelastic deformation mechanism of the flash-sintered YSZ changes prominently. Therefore, Zone 1 in Fig. 3i corresponds to transformation toughening-dominated region below 400 °C; whereas, Zone 2 is associated to dislocation activity and presumably grain boundary sliding dominant region above 400 °C.

An indirect way to confirm martensitic transformation is to check the existence of reverse transformation^9^. As shown in Fig. 4a–e, a significant amount of recoverable strain was observed during partial unloading owing to reverse transformation at room temperature. Furthermore, hysteresis loops appear as strain increases. The area of the hysteresis loop tends to increase as stress and strain both increase. The appearance of hysteresis loops may be attributed to reverse transformation and reopening of closed pores in the flash-sintered 3YSZ^49^. The pillar after the cyclic loading test experiences 1% plastic strain, but still does not exhibit cracks as can be seen in Fig. 4b. It is worth mentioning that the stress–strain curves show stronger linearity as the cyclic compression tests are conducted on the same pillar. The stress–strain curve for the second monotonic test shows the highest linearity compared to the cyclic loading and the first monotonic compression test. This implies that martensitic transformation begins to concede as consecutive compression tests are carried out on the same pillar because part of the transformed monoclinic phase does not revert to tetragonal as the load on the pillar is removed^50^. Cyclic loading tests at 400 °C show significantly less recoverable strain than the room temperature tests due primarily to the lack of transformation toughening, but the hysteresis loop remains visible presumably due to reopening of the microcracks and pores. An increase of crack density and slow crack propagation seem to prevent the pillars from undergoing brittle fractures and the pillar achieves a residual strength of 1.5 GPa. Furthermore, stress relaxation measured during the holding segment (1 s for cycles) is observed at both room temperature and 400 °C (Supplementary Fig. 12). Room temperature test exhibits the same amount of stress relaxation during holding regardless of the overall strain. The formation of new microcracks and strain energy reduction in the pillar may result in the stress relaxation at room temperature^51^. On the other hand, a significant increase of stress relaxation (the absolute value of stress reduction) is observed at 400 °C and the stress relaxation increases as the pillar undergoes plastic flow. Stress relaxation at high temperature is normally due to high temperature and stress-induced sintering and closure of pores^51^. However, this mechanism is less likely at the low temperature (400 °C). Thus, stress relaxation taking place at 400 °C may be mainly contributed by a thermally activated inelastic process such as the effect of grain boundary sliding, and/or diffusional creep of dislocations generated during the ultrafast sintering process. Another possible explanation is transient dislocation movement during the holding segment triggered by dislocation gliding, which is more likely to happen at elevated temperatures^28^. Thus, the observation of stress relaxation may indicates that the dislocation activity contributes to deformability of flash-sintered 3YSZ together with grain boundary sliding and/or diffusion-induced inelastic mechanism above 400 °C. The proposed relaxation mechanisms are all thermally activated phenomenon and time is an important variable. To better understand the inelastic mechanism activated at elevated temperature, systematic strain rate jump microcompression tests are needed in future studies.

In conclusion, the first in situ study on mechanical behavior of flash-sintered YSZ was performed inside SEM at elevated temperatures up to 600 °C. At room temperature, YSZ micropillars sustain giant strain (~8%) comparing with its bulk counterpart (~2%) due to the stress-induced martensitic transformation toughening. However, the pillars fracture catastrophically after the nucleation of cracks. In comparison, a brittle-to-ductile transition of fracture mode is observed at 400 °C in flash-sintered YSZ, much lower than the ~800 °C reported in conventional bulk YSZ. The enhanced plasticity at elevated temperatures arises from the transition from phase transformation toughening to dislocation creep, as the dominant inelastic deformation mechanism due to the existence of a high density of dislocations in flash-sintered YSZ and/or to early initiation of grain boundary sliding of ultra-fine grains. This study provides the first evidence on superior mechanical properties of flash-sintered ceramics by using in situ nanomechanical testing technique at elevated temperatures and establishes an important way to fundamentally understand the densification and mechanical properties of ceramics.

## Methods

### Flash sintering

Flash sintering was performed on a custom-modified thermomechanical testing system (SETSYS Evolution, SETARAM Instrumentation). Specimens with a diameter of 5 mm and a thickness of 2 mm prepared by using commercially available 3YSZ (TZ-3Y-E, Tosoh corp., 40 nm particle size) were sandwiched between two platinum electrodes. An alumina rod was utilized to apply minimum pressure (a few kPa) to ensure rigid contact between the electrodes and sample. A DC power of various voltage was applied to achieve electric field of 1.5, 15, and 150 V/cm with a constant heating rate of 25 °C/min (maximum temperature was set to 1300 °C). The experiment was performed in the presence of air. After the onset of flash, the system was switched from the voltage-control mode to a current-control mode. The experiment was terminated right after switching to current-control mode to prohibit grain growth. The linear shrinkage of the samples was measured by a dilatometer.

### TEM sample preparation

Plan-view TEM samples of flash-sintered 3YSZ were prepared through the conventional approach, which includes manual grinding, polishing, dimpling, and final polishing in an ion milling system (PIPS II, Gatan). Low energy ion polishing (2 kV) was used to minimize ion milling-induced damage. An FEI Talos 200X TEM/STEM with ChemiSTEM technology (X-FEG and SuperX EDS with four silicon drift detectors) operated at 200 kV was used in this study for microstructure characterization and energy-dispersive X-ray spectroscopy (EDS) chemical mapping.

### Microcompression test

Micropillars of flash-sintered 3YSZ with ~3 µm in diameter and a diameter-to-height aspect ratio of 1:3–1:2 were prepared using focused ion beam (FEI quanta 3D FEG) and a series of concentric annular milling and polishing with progressively de-escalated currents were adopted to reduce tapering angle. Micropillar compression experiments have been performed using a Hysitron PI 88×R PicoIndenter equipped with a piezoelectric actuator on the capacitive transducer that enables the collection of force–displacement data inside a scanning electron microscope (FEI quanta 3D FEG). Moreover, a 20 µm diamond flat punch tip designed for high-temperature compression experiments was used to conduct in situ compression experiments and the geometric variation of micropillars was synchronized to evolving force–displacement curve. For high-temperature in situ compression setups, the flat punch was fastened to probe heater and the specimens were clamped by a V-shaped molybdenum to a ceramic heating stage. The temperature on two heating terminals were simultaneously ramped up at a rate of 10 ˚C/min and isothermally preserved for 3 min before implementing every single compression experiment to eliminate the thermal-driven drifts on both probe and stage sides. An average drift rate of 0.2–0.5 nm/s was estimated in preloading process for 45 s and the estimated force noise level is less than 8 µN prior to compression. An overestimation of specimen displacement during the compression test induced by a displacement associated with the measuring instrument (machine compliance) was systematically measured during in situ SEM studies and corrected.

### Finite-element analysis

The microstructure shown in Supplementary Fig. 3a with three different grain orientations was subjected to a compression of −200 MPa in the in-plane vertical direction, while the bottom boundary was fixed, and the out-of-plane state of stress was set to plane strain conditions. Material properties are summarized in Table 1. Assigned Euler angle orientations are, for the bottom grain, (α, β, γ) = (0,0,0), for the top-right grain, (*α*, *β*, *γ*) = (0, 45, 0) and for the top-left grain, (*α*, *β*, *γ*) = (45,45,0). Euler angles operations are: first rotate angle *β* degrees about the *z*-axis, then rotate *α* degrees about the *y*-axis, and finally rotate *γ* degrees about new *z*-axis. The mechanical equilibrium state of the polycrystalline was solved using OOF2^52^, an open source implementation of the finite-element method. The relative numerical tolerance was set to 1 × 10^−10^. The simulation used 40 GB of RAM and a wall time of ~4 h (See Supplementary Methods for detailed methodology).

**Table 1:** Elastic properties of tetragonal zirconia^46^

C_11_ (GPa)	C_12_ (GPa)	C_13_ (GPa)	C_33_ (GPa)	C_44_ (GPa)	C_66_ (GPa)
395	26	105	326	42	56

### Data availability

The data that support the findings of this study are available from the corresponding author on reasonable request.

## Electronic supplementary material

Supplementary Information

Description of Additional Supplementary Files

Supplementary Movie 1

Supplementary Movie 2

Supplementary Movie 3
