# Supplementary material for: High temperature deformability of ductile flash-sintered ceramics via in-situ compression
Source: Nat Commun. 2018 May 25;9:2063. doi: 10.1038/s41467-018-04333-2 (PMC5970235; doi:10.1038/s41467-018-04333-2)
Supplement: Supplementary file 1 — Supplementary Information [file 41467_2018_4333_MOESM1_ESM.pdf]

Supplementary Information

High temperature deformability of ductile flash sintered ceramics via *in-situ* compression

Cho et al.

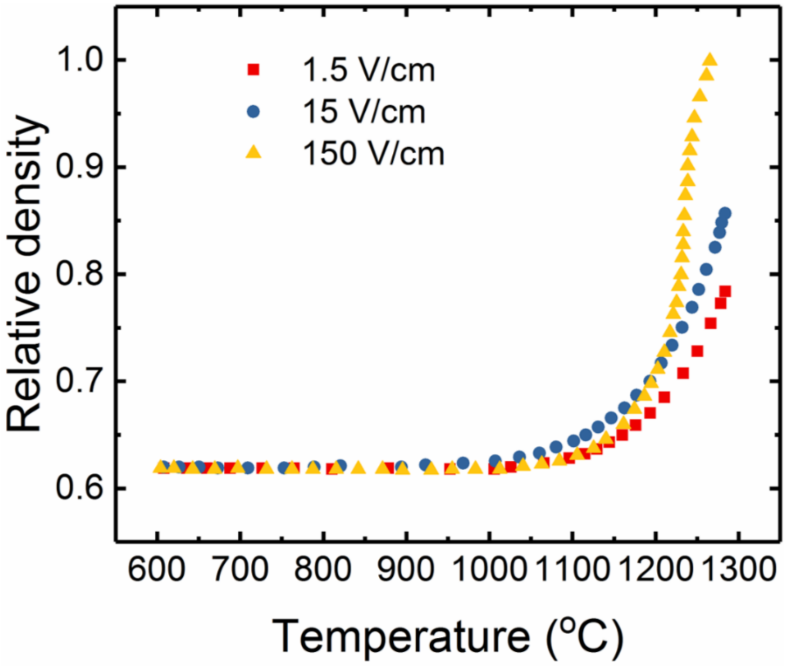

Supplementary Figure 1. Relative density comparing to theoretical value as a function ramping temperature during flash sintering. 3YSZ was sintered at a constant heating rate of 25°C/min under an electrical field of 1.5, 15, and 150 V/cm. Flash sintering under an electric field of 150 V/cm took place at 1,150-1,200^{o}C in a few seconds.

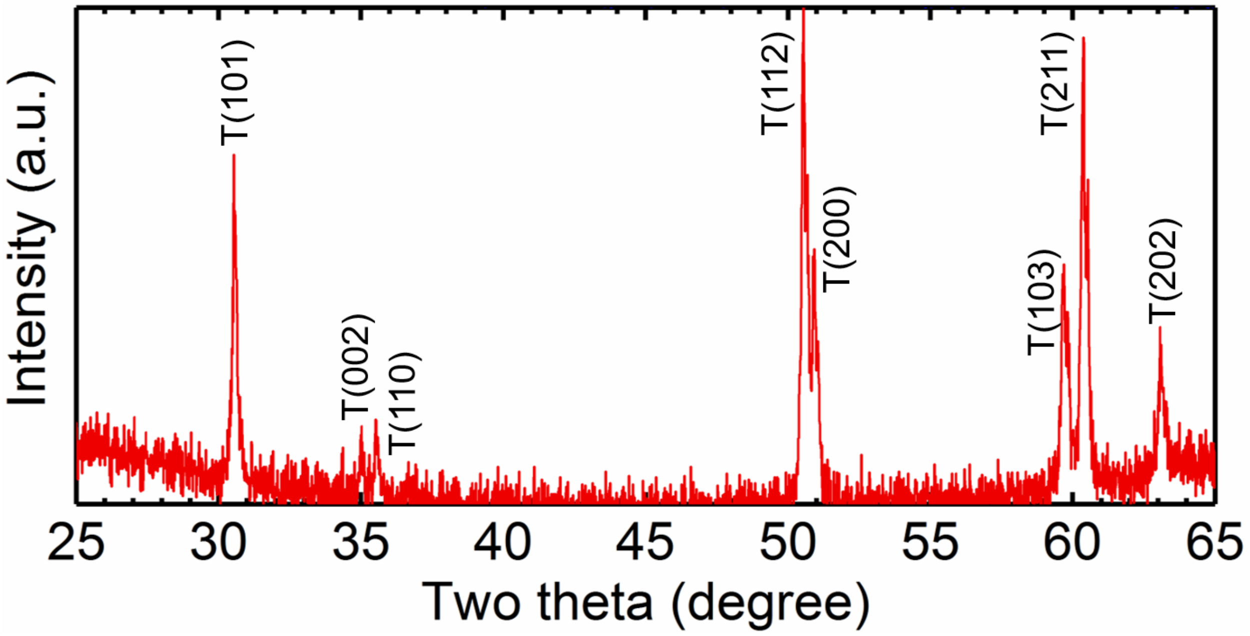

Supplementary Figure 2. XRD patterns of flash sintered 3YSZ at an electrical field of 150 V/cm, showing dominant metastable tetragonal phase and crystallographic plane indices.

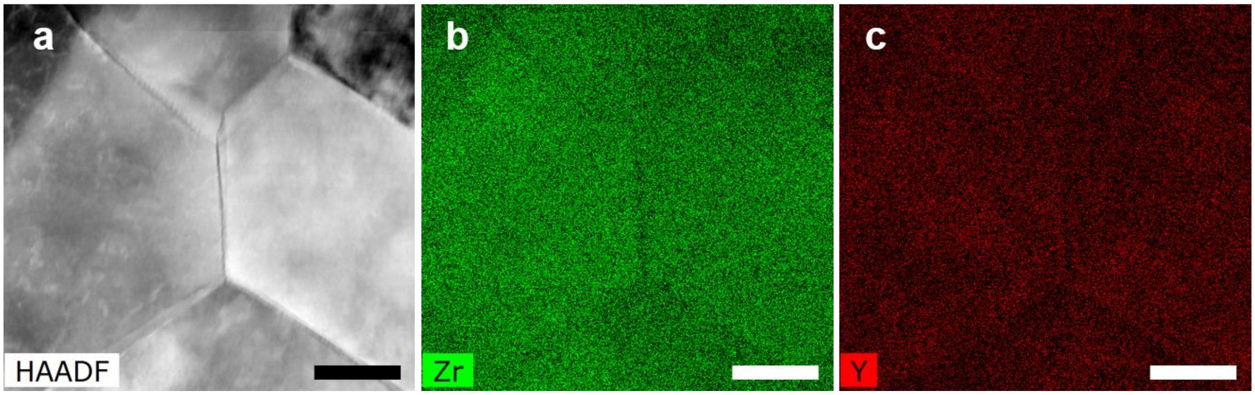

Supplementary Figure 3. STEM micrograph. **a** A STEM micrograph of the flash-sintered 3YSZ showing several triple junctions among grains. Scale bar, 60 nm. **b-c** The EDS mapping of zirconium and yttrium. Note that zirconium and yttrium are nearly uniformly distributed throughout the grains. The grain boundaries appear to have slight deficiency in zirconium. Scale bar, 60 nm.

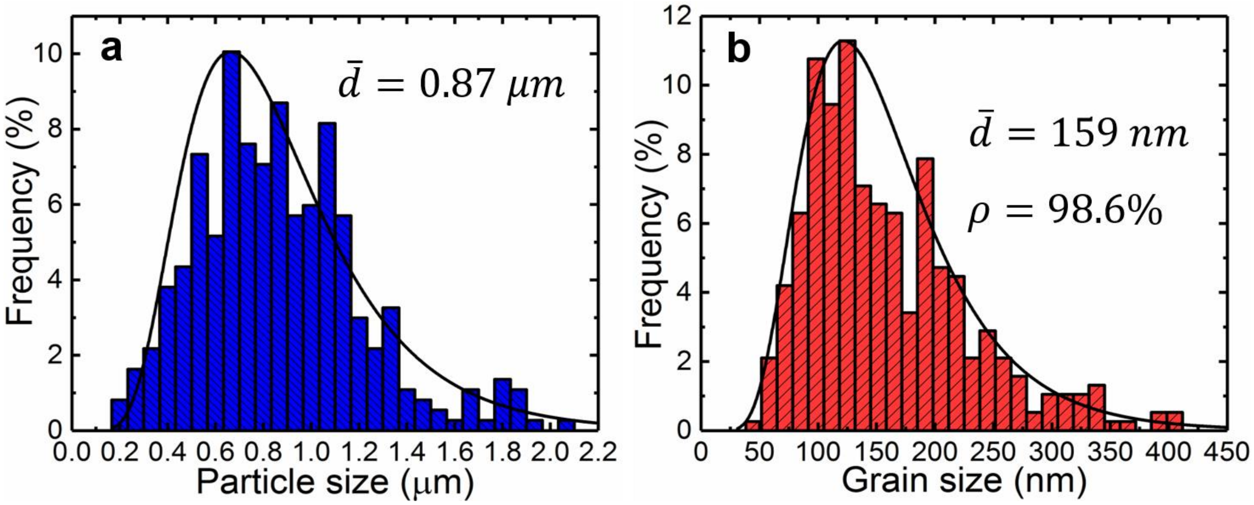

Supplementary Figure 4. Grain size distribution. **a** Agglomerated particle size distribution and **b** subgrain size distribution of flash- sintered 3YSZ calculated by systematic grain intercept method. The average grain size and subgrain size are 0.87 μm and 159 nm, respectively. The density of flash-sintered ceramic is ~ 98%.

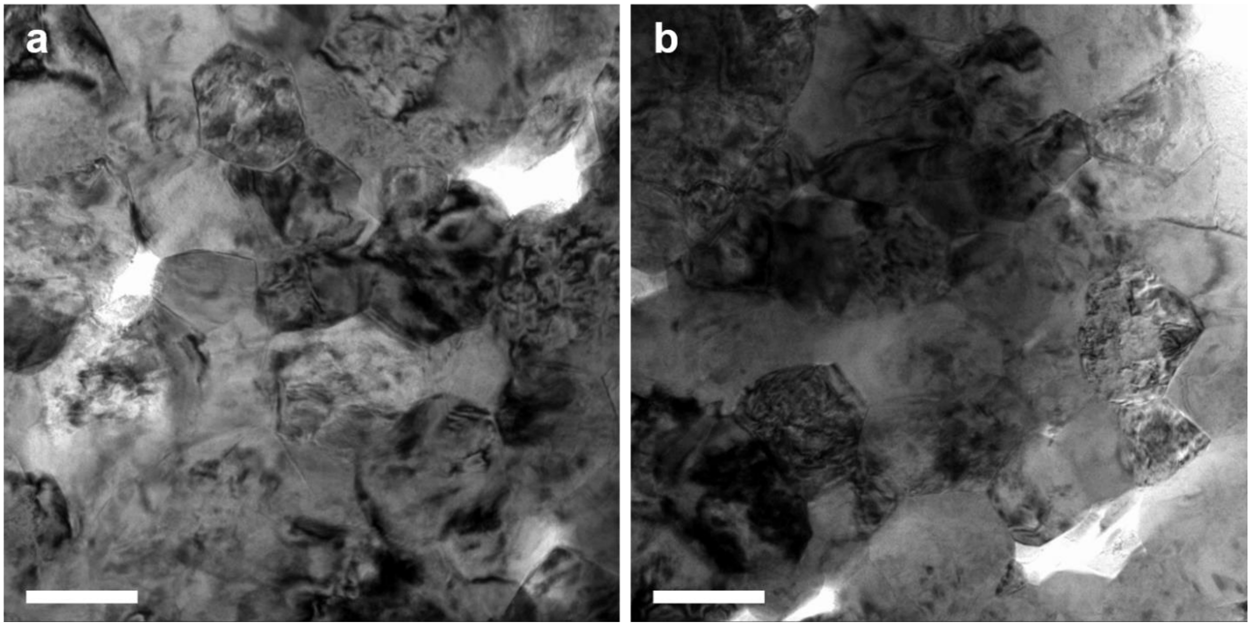

Supplementary Figure 5. Bright-field TEM images of flash-sintered 3YSZ under an electric field of 150 V/cm. **a-b** TEM images taken from different regions showing the existence of grains with significant internal defects. Many of these internal defects appear to be high-density dislocation networks. Scale bar, 200 nm.

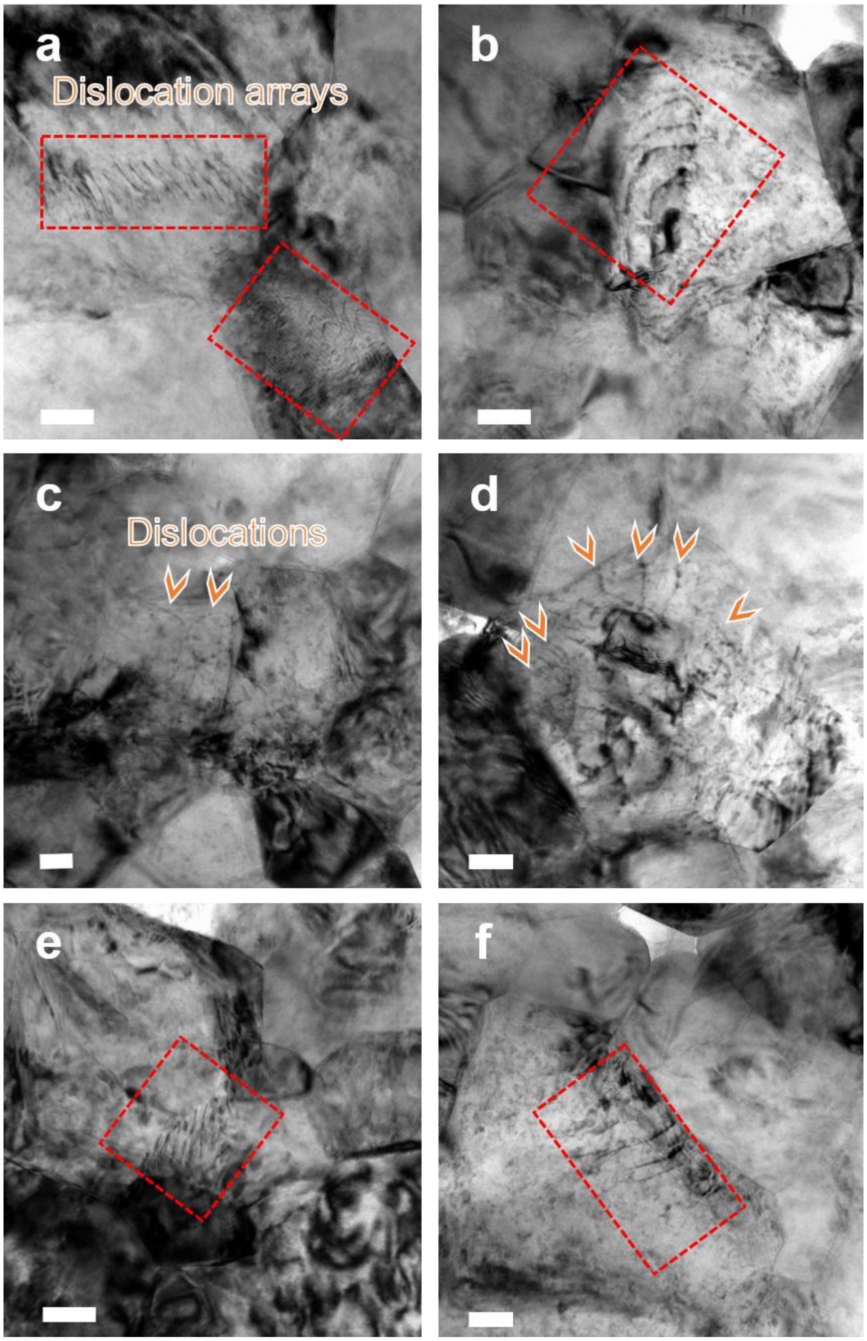

Supplementary Figure 6. Bright-field TEM micrographs. **a-f** Bright-field TEM images of the flash sintered 3YSZ showing the existence of dislocations and dislocation arrays in grains. Dislocation arrays were frequently observed near triple junctions. Dislocations may arise from the large stress concentration near triple junctions induced during flash-sintering of 3YSZ. Scale bar, 50 nm.

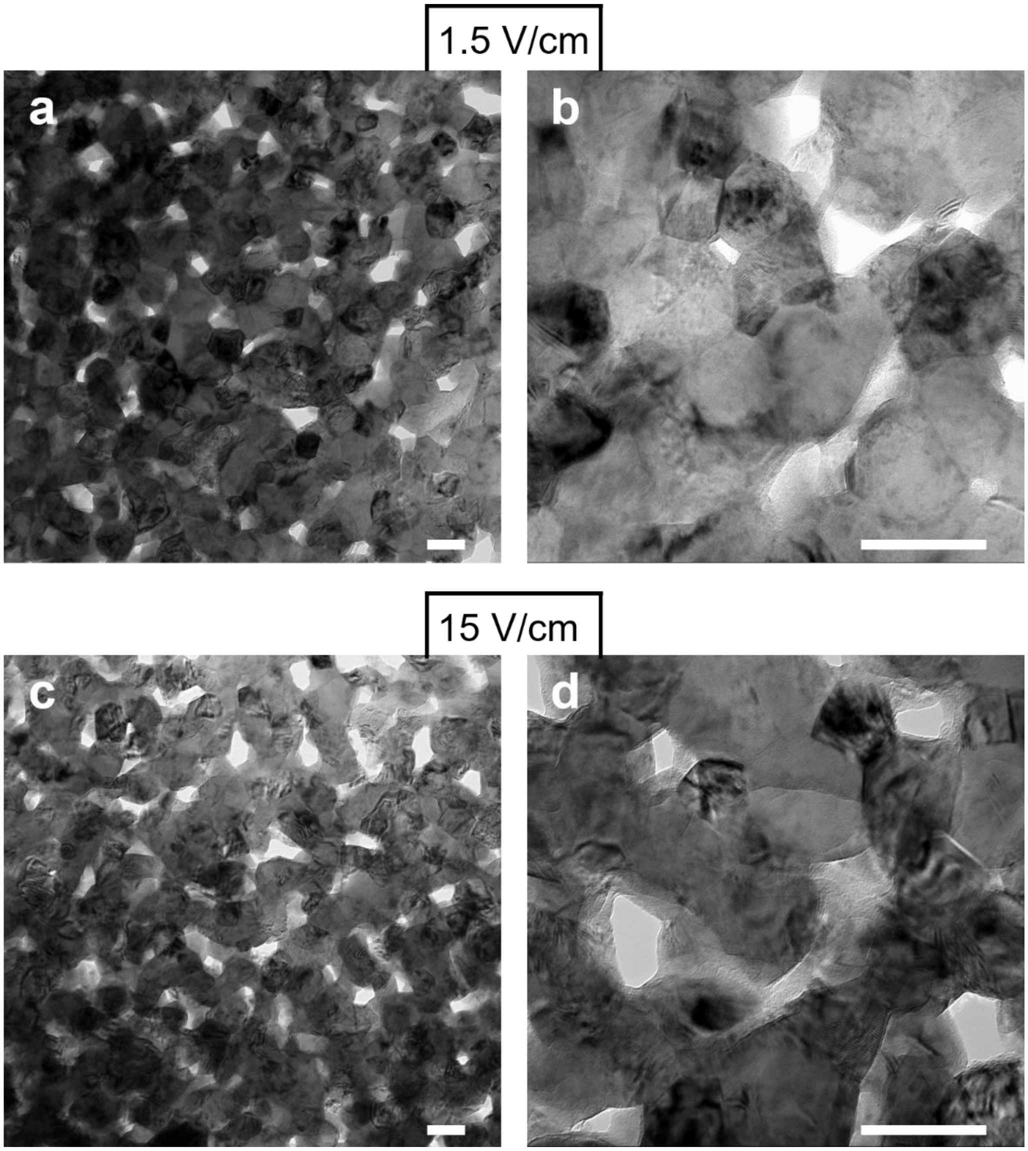

Supplementary Figure 7. Bright field TEM images of 3YSZ processed under 1.5 V/cm and 15 V/cm showing nanograins. **a-b** 3YSZ sintered under 1.5 V/cm. Scale bar, 100 nm. **c-d** 3YSZ sintered under 15 V/cm. Scale bar, 100 nm. Dislocations and dislocation arrays rarely exist inside the grains unlike 3YSZ sintered under 150 V/cm.

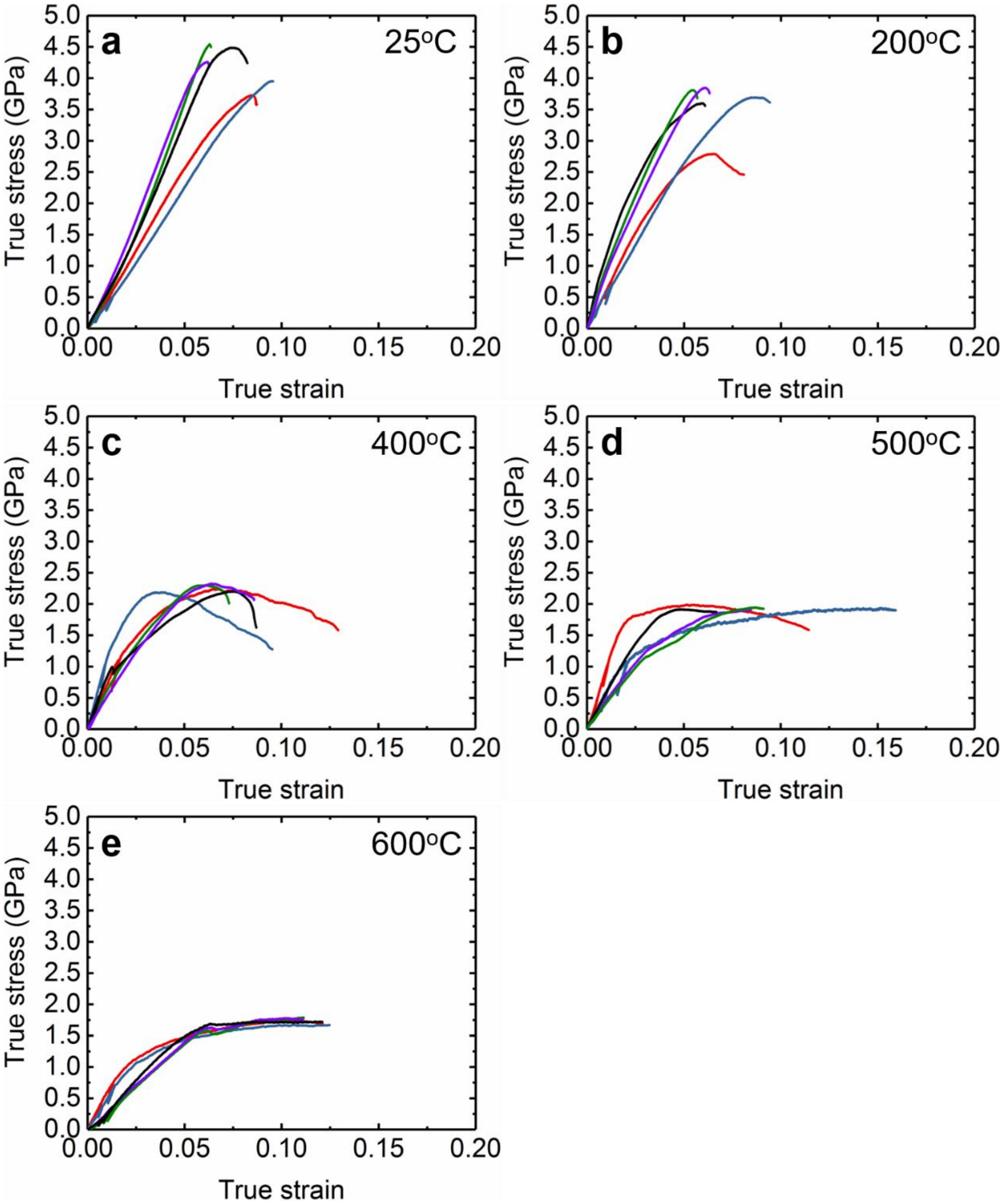

Supplementary Figure 8. Repeatability of true stress-strain curves for 3 YSZ tested at **a** 25°C, **b** 200°C, **c** 400°C, **d** 500°C, and **e** 600°C. Five microcompression tests were carried out to check repeatability of the tests. Flow stresses in most studies show good agreement. Unloading studies at 0.5 and 1% strain were carried out to measure the apparent elastic moduli at each temperature.

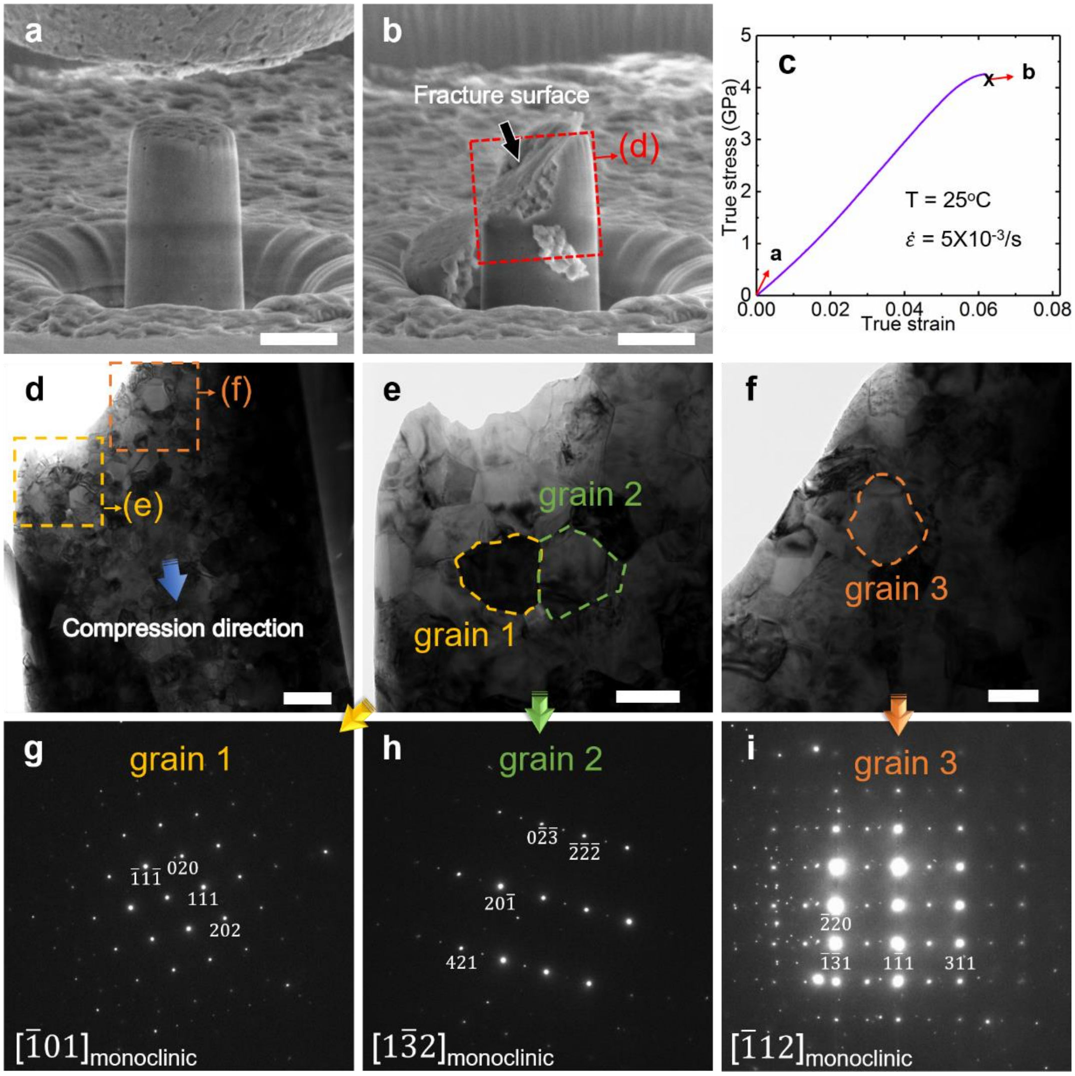

Supplementary Figure 9. TEM analyses of a 3YSZ pillar tested at room temperature. **a-b** The SEM image of the pillar before and after compression test at room temperature. Scale bar, 2 µm. **c** The corresponding stress-strain curve showing a flow stress of 4 GPa and a fracture strain of ~ 6%. **d** Bright-field field XTEM image of the fractured pillar. Scale bar, 500 nm. **e-f** Bright-field XTEM images of the pillar showing ultrafine grains near fracture surface. Grains 1-3 were chosen to identify phase transformation. Scale bar, 200 nm. **g-i** Selected area electron diffraction (SAED) patterns showing that grains 1-3 have monoclinic zirconia phase examined along respective zone axis of $[\bar{1}01]$, $[1\bar{3}2]$, and $[\bar{1}12]$.

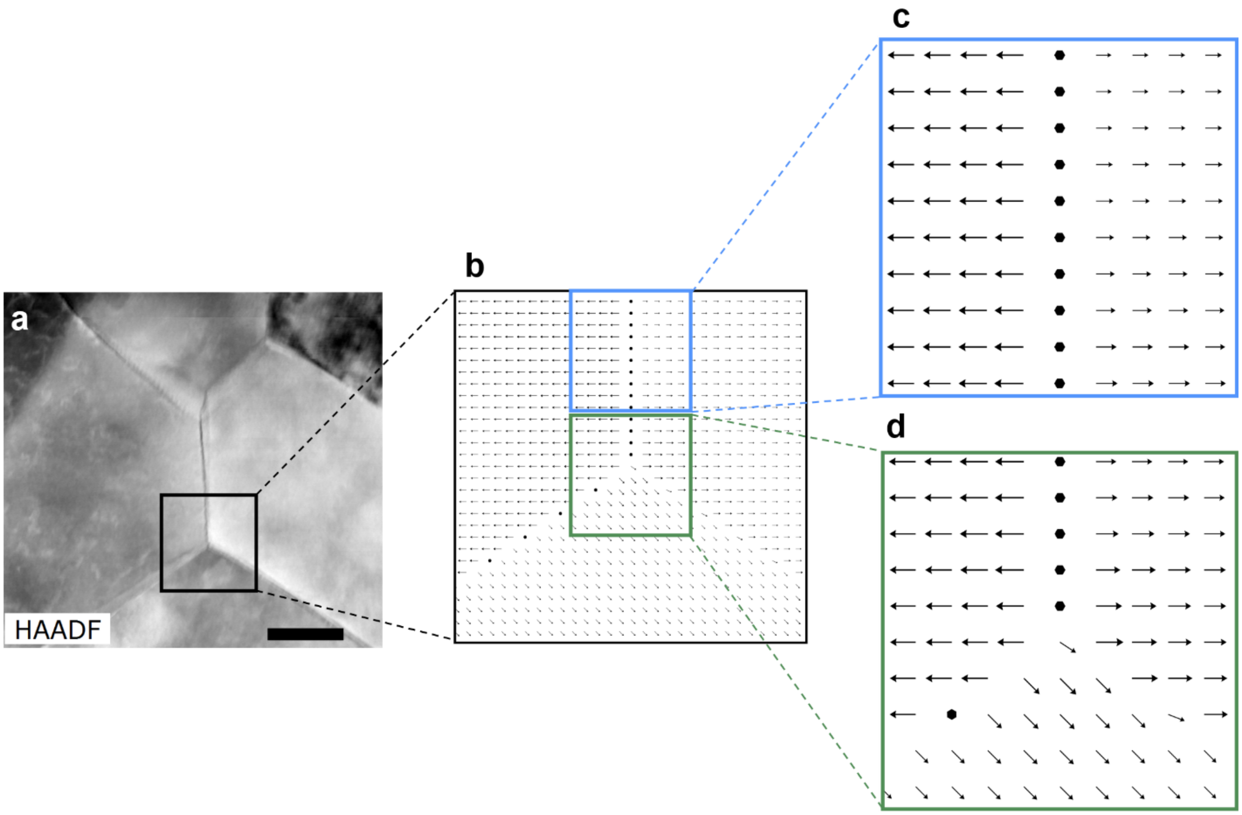

Supplementary Figure 10. Peach-Köhler force distribution in the vicinity of grain boundaries. **a** Original microstructure. Scale bar, 60 nm. **b** Zoom-in region around a selected grain corner highlighted in the black-box region. Assigned Euler angle orientations are, for the bottom grain (α, β, γ) = (0,0,0), for the top-right grain, (α, β, γ) = (0, 45, 0) and for the top-left grain, (α, β, γ) = (45 ,45,0). For the BCT system, $\vec{b} = 3.16[\bar{1}11]$ Å and dislocation line, $\vec{\xi} = [110]$[3], **c-d** The Peach-Köhler forces for a polycrystal subjected to a −200MPa in-plane vertical compression for plane strain conditions at selected locations. The maximum magnitude of the force per unit length on the test dislocations is |F|=0.059N/m. For this particular Burgers vector and line direction, the resultant force field favors dislocations to pile up in the abutting grains, making grain boundaries and corners sources and sinks of dislocations.

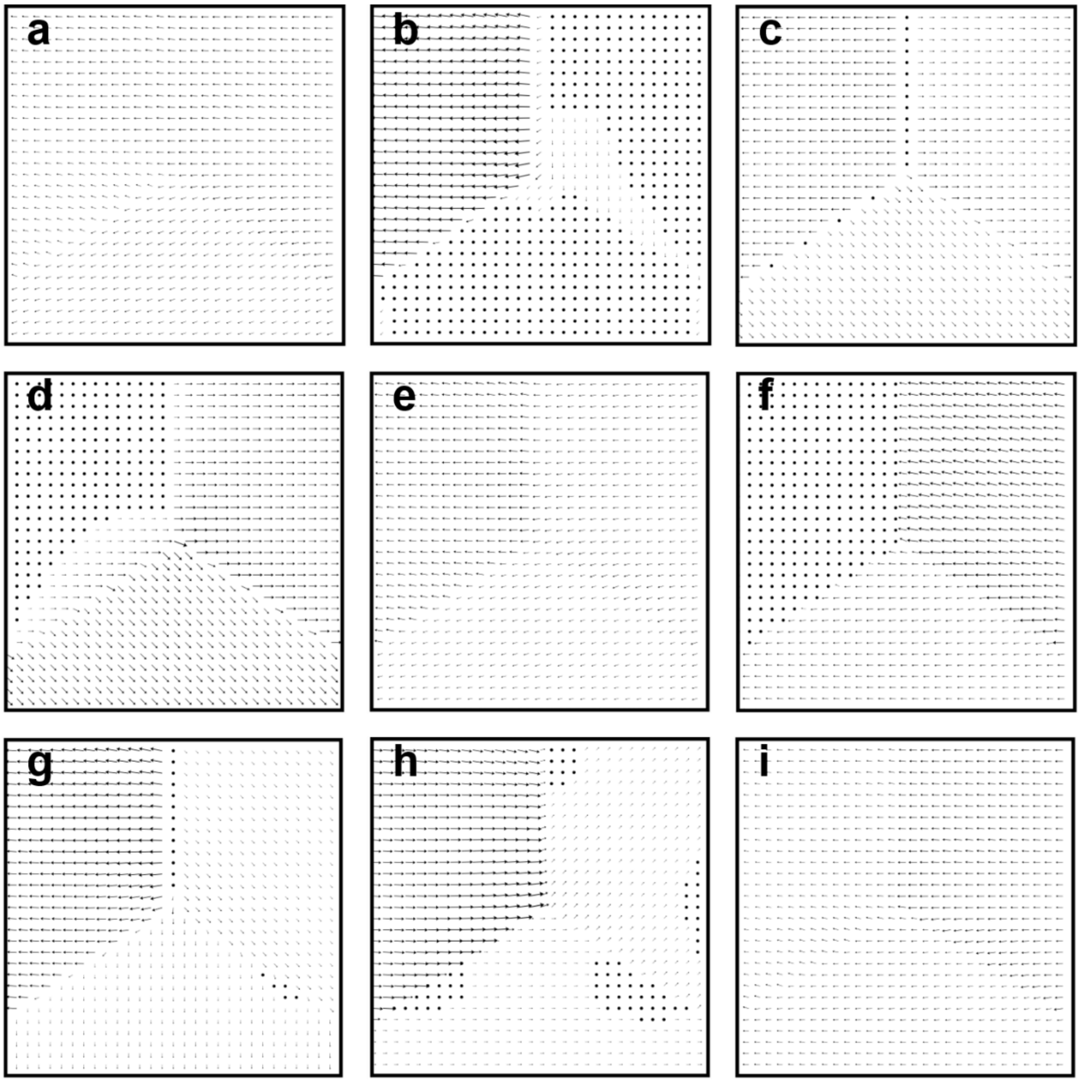

Supplementary Figure 11. Summary of Peach-Köhler forces for microstructure shown in Supplementary Fig. 10a. Each inset shows a different test dislocation line: **a** $\vec{b} = 3.16[111]$Å and $\vec{\xi} = [111]$, **b** $\vec{b} = 3.16[111]$Å and $\vec{\xi} = [1\bar{1}0]$, **c** $\vec{b} = 3.16\,[\bar{1}11]$Å and $\vec{\xi} = [110]$, **d** $\vec{b} = 3.16[111]$Å and $\vec{\xi} = [110]$, **e** $\vec{b} = 3.16[111]$Å and $\vec{\xi} = [101]$, **f** $\vec{b} = 3.16[111]$Å and $\vec{\xi} = [011]$, **g** $\vec{b} = 3.16[111]$Å and $\vec{\xi} = [100]$, **h** $\vec{b} = 3.16[111]$Å and $\vec{\xi} = [010]$, and **i** $\vec{b} = 3.16[111]$Å and $\vec{\xi} = [001]$. In all cases, grain corners and boundaries become sources or sinks for dislocations.

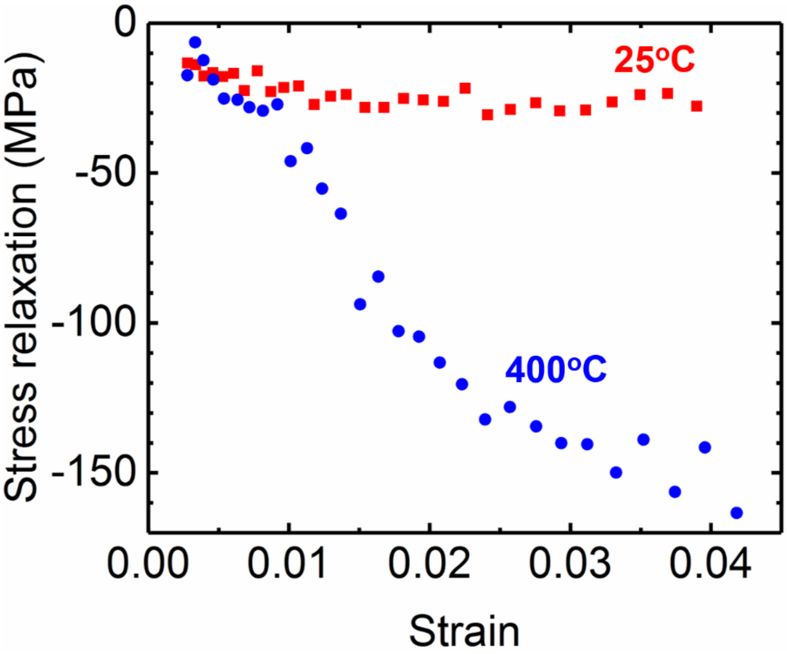

Supplementary Figure 12. Stress relaxation as a function strain at room temperature and 400°C during one second holding segment of cyclic loading and unloading tests.

Supplementary Methods

Finite element method analysis on stress field distribution for dislocations. The effects of an externally applied or self-induced stress field, on polycrystalline YSZ was determined by solving the mechanical equilibrium equation:

$$\nabla \cdot \overleftrightarrow{\sigma} = \vec{0} \tag{1}$$

where the elastic stress, $\overleftrightarrow{\sigma}$, is linearly proportional to the elastic strain, $\overleftrightarrow{\varepsilon}$, as specified by Hooke's law, $\sigma_{ij} = C_{ijkl}\varepsilon_{kl}$. The resultant state of stress distribution in a polycrystal imposes a Peach-Köhler force on any existing dislocations, $\vec{F} = (\vec{b} \cdot \overleftrightarrow{\sigma}) \times \vec{\xi}$, which in turn induces dislocation motion, i.e., $\vec{v} = M\vec{F}$ [1]. For YSZ, nine crystallographically possible elemental dislocation configurations were numerically explored through finite element method (FEM) analysis[2]. Overall, calculations demonstrate that shear stresses concentrate at grain corners and boundaries and enable the nucleation and propagation of dislocations into the grains, thus promoting plastic deformation and leading to the pile-up of defects at the core of the abutting grains. An example of these calculations is shown in Supplementary Fig. 10 for an arbitrary crystallographic orientation of grains assigned to the microstructure of Supplementary Fig. 3a. For $\vec{b} = 3.16[\bar{1}11]$ Å and $\vec{\xi} = [110]$[3], line defects are favored to move away from the grain boundary into the grain. At the grain boundary, Supplementary Fig. 10c shows outward pointing forces on both sides of the grain boundary, pulling dislocations into the grain(s), while enabling metastable dislocation configurations at the grain boundary. Similarly, Supplementary Fig. 10d shows that grain corners act as a dislocation source, greatly contributing to the plastic deformation of the surrounding grains. In some cases, the extent of the forces exerted on the dislocations only spans a few nanometers and, in other cases, the same grain boundary can act as

a dislocation sink on one side of the interface and as a source on the other. Finally, in some cases, dislocations are entirely unaffected by the grain boundary, but the grain corner always remains active (Supplementary Fig. 11). These effects are more pronounced for large angle misorientations, and stresses above the yield point.

Supplementary References

1. Peach, M. & Köhler, J. S. The forces exerted on dislocations and the stress fields produced by them. *Phys. Rev.* **80**, 437-439 (1950)

2. Reid, A. C. E. et al. Modelling microstructures with OOF2. *Int. J. Mater. Prod. Tec.* **35**, 361-373 (2009)

3. Zhao, X. S., Shang, S. L., Liu, Z. K. & Shen., J. Y. Elastic properties of cubic, tetragonal and monoclinic ZrO_2 from first-principles calculations. *J. Nucl. Mater.* **415**,13-17 (2011)
