# Supplementary material for: High temperature deformability of ductile flash-sintered ceramics via in-situ compression
Source: Nat Commun. 2018 May 25;9:2063. doi: 10.1038/s41467-018-04333-2 (PMC5970235; doi:10.1038/s41467-018-04333-2)
Supplement: Supplementary file 2 — Description of Additional Supplementary Files [file 41467_2018_4333_MOESM2_ESM.pdf]

Description of Additional Supplementary Files

File Name: Supplementary Movie 1
Description: A video showing a typical in-situ microcompression test on the flash sintered 3YSZ at 25o C performed in SEM. Note that the pillar can accommodate true strain as much as 8% before it experiences catastrophic failure.

File Name: Supplementary Movie 2
Description: A video showing in-situ microcompression test on the flash sintered 3YSZ at 400o C in SEM. Note that 400o C is a demarcation temperature where a new inelastic deformation mechanism begins to operate. Early crack occurrence at low strain due to the lack of transformation-induced toughening was observed.

File Name: Supplementary Movie 3
Description: A video showing in-situ microcompression test on the flash-sintered 3YSZ performed at 600o C in SEM. Note that the pillar can accommodate 15% of true strain at the test temperature without catastrophic failure.
